# Biofeedback Therapy in Managing Functional Fecal Incontinence in Children: A Literature Review

**DOI:** 10.7759/cureus.74295

**Published:** 2024-11-23

**Authors:** Sara Caldas Afonso, Nuno Caria Ramalhao, Ana Cavalheiro, Ana Trepa

**Affiliations:** 1 Physical Medicine and Rehabilitation, Centro Hospitalar Universitário de Santo António, Porto, PRT

**Keywords:** biofeedback therapy, functional constipation, functional fecal incontinence, functional nonretentive fecal incontinence, pediatric rehabilitation

## Abstract

This literature review explores the role of biofeedback therapy (BFT) in managing functional fecal incontinence (FFI) in children - a common condition with a substantial impact on the quality of life. FFI diagnosis relies primarily on medical history and thorough physical examination and is categorized by the Rome IV criteria into functional constipation (FC) and functional nonretentive fecal incontinence (FNRFI). Treatment options for FFI remain limited, particularly for FNRFI. BFT employs electronic or mechanical devices, such as rectal probes or surface electrodes, to provide real-time feedback on muscle activity and rectal pressure. This feedback allows patients to better understand and control their pelvic floor muscles, improving coordination between contraction during stool retention and relaxation during defecation. It also plays a role in rectal sensory awareness, enabling patients to respond appropriately to the urge to defecate. BFT has been considered an option in refractory cases, although evidence supporting its routine use is still emerging. We conducted a comprehensive literature search focusing on studies from the past 24 years that evaluated BFT for pediatric FFI. Five relevant studies were identified and analyzed, each utilizing BFT in combination with various treatment modalities. Two studies, both randomized controlled trials (RCT) and with the largest sample, focused on the treatment of FNRFI, both concluding that BFT should be used in FNRFI refractory to conventional treatment. Another two studies, one RCT and a retrospective study, focused on patients with FC. The first did not show any additional value in the use of BFT, while the second showed positive results. Comparing both studies, they had very different methodologies and treatment plans, but besides these results, in both studies, they concluded that when selecting a treatment plan for a child with FFI, an alternative or additional treatment with BFT should be considered. Finally, the last study, a quasi-experimental study, did not differentiate between FC or FNRFI and compared the use of BFT to percutaneous tibial nerve stimulation (PTNS). Although they showed that there were slightly better results when using PTNS, they concluded that both PTNS and BFT are effective modalities in treating children with FFI in addition to conventional treatment. Overall, BFT showed positive outcomes, with no safety issues reported. BFT appears to be a useful, non-invasive option for pediatric FFI, both FC and FNRFI, especially in cases unresponsive to conventional therapies. When used in a tailored, multimodal approach, BFT holds the potential to improve continence and quality of life in children with this challenging condition, and given that 15% of children with FFI, specifically FNRFI, continue to experience symptoms into adulthood, it is crucial to consider these treatment options early to potentially reduce this rate. Besides this, more research is needed to conclude the long-term effects and to establish standardized pediatric rehabilitation protocols.

## Introduction and background

Fecal incontinence 

Introduction and Epidemiology

Fecal incontinence (FI) in children is characterized by the involuntary passage of stool in socially inappropriate settings at least once a month in children with a developmental age of four years or older [1,2]. This condition presents significant challenges for both children and their parents, often resulting in feelings of embarrassment and guilt. These emotions can make children more vulnerable to bullying and social rejection, potentially leading to social withdrawal and avoidance of school. Consequently, FI has a profound impact on the quality of life for affected children [3,4]. FI can be classified as either organic or functional. Organic FI includes cases associated with underlying conditions, such as repaired anorectal malformations, post-surgical Hirschsprung’s disease, spinal dysraphism, spinal cord injuries, cerebral palsy, myopathies that affect the pelvic floor and external anal sphincter, amongst many others. However, 95% of cases are classified as functional, which can be further divided into FI associated with constipation (FC) and functional non-retentive fecal incontinence (FNRFI), as described by the Rome IV criteria, as can be seen in Table 1 [1]. Among functional cases, approximately 80% are attributed to FC, with the remaining 20% classified as FNRFI [4]. Prevalence data for FI in children varies depending on age and study parameters. FC prevalence ranges from 0.7% to 29.6%, while FNRFI is estimated to affect about 1% of the general pediatric population, with recent global data reporting a pooled prevalence of approximately 0.4% [5,6]. 

**Table 1:** Rome IV criteria for functional constipation and functional nonretentive fecal incontinence. Adapted from Ref. [1]

Functional nonretentive fecal incontinence	Functional constipation
Must fulfill all of the following for ≥1 month prior to diagnosis in a chilld with a developmental age of at least four years:	Must fulfill ≥2 criteria at least once per week for a minimum of one month prior to diagnosis, with insufficient criteria for the diagnosis of irritable bowel syndrome:
1. Defecation into places inappropriate to the social context at least once per month	1. ≤2 defecations in the toilet per week in a child of a developmental age of at least four years
2. No evidence of fecal retention	2. At least one episode of fecal incontinence per week
3. After appropriate medical evaluation, the fecal incontinence cannot be explained by another condition	3. History of retentive posturing or excessive volitional stool retention
	4. History of painful or hard bowel movement
	5. Presence of a large fecal mass in the rectum
	6. History of large-diameter stools, which may obstruct the toilet

Pathophysiology 

FC is largely associated with inadequate toilet training and withholding behaviors that trigger physiological changes promoting stool retention and fecal mass accumulation. As water is reabsorbed from the retained feces, the stool hardens, making it more challenging to expel and reducing rectal sensitivity. Consequently, stools from the upper portion of the mass liquefy due to bacterial activity, seeping past the hardened fecal mass and causing incontinence [2-4]. By contrast, the precise pathophysiological mechanisms behind FNRFI remain unclear. Studies suggest that children with FNRFI typically exhibit normal anorectal function. One hypothesis is that these children may habitually ignore or delay the urge to defecate to avoid interrupting activities such as playing video games [3,7]. Other suggested contributing factors include significant life changes, such as parental divorce, the birth of a sibling, or other psychological stressors [7,8].

Diagnosis

The diagnosis of FFI primarily relies on a comprehensive medical history and a thorough physical examination, including abdominal, anorectal, and neurological assessments. Imaging or additional diagnostic tests are generally unnecessary; however, in cases where the diagnosis is uncertain or the patient’s history is inconsistent, further evaluations such as colonic transit studies, anorectal manometry, or colonic manometry may be considered [4,9].

Table 2 provides a comparative overview of the characteristics distinguishing FC from FNRFI.

**Table 2:** Comparison between functional constipation and functional nonretentive fecal incontinence. Adapted from Ref. [4]

Clinical and imagiologic feature	Functional constipation	Functional nonretentive fecal incontinence
Nature of stools	Hard and lumpy	Normal formed stools
Amount of incontinent stools	Small amounts of either liquid or formed stools	Regular or usual amount of stools
Stool frequency	<2 stools per week	Normal frequency of stools
Large amount of stool	Frequent	Never
Pain during defecation	Frequent	Rare
Daytime fecal incontinence	Frequent	Always
Nighttime fecal incontinence	Frequently in severely constipated children	Never
Colonic transit time	Usually prolonged	Normal
Anorrectal manometry	High sensory threshold/dyssynergic defecation	Normal
Colonic manometry	Low frequency of high-amplitude propagatory contractions	Normal

Treatment and Biofeedback Therapy 

The primary goal in treating FI is to achieve normal continence with regular, predictable bowel movements [10]. Effective treatment of FI requires a multimodal approach, often beginning with non-pharmacological strategies. These include educating both the child and parents about the condition, implementing strategies such as toilet training, maintaining a bowel diary, and possibly using a reward system to encourage compliance [11-13]. Pharmacological interventions, including laxatives, enemas, and rectal irrigation, are typically reserved for cases involving FC as there is insufficient evidence supporting their use in FNRFI [14,15]. Additional therapeutic techniques, such as electrostimulation, pelvic floor exercises, tibial nerve stimulation and BFT, may be incorporated.

Relating to BFT, it uses electronic and mechanical devices to enhance the neuromuscular system's ability to coordinate and execute the processes involved in continence, defecation, and also in sensory rectal awareness. It achieves this by providing patients with real-time feedback on their physiological responses, allowing them to consciously modify and improve these functions over time [11-12,16]. This can be achieved either through electromyography (EMG) using surface electrodes to monitor muscle activity or through pressure sensors that detect changes in anorectal pressure. The aim is to improve the squeeze pressure of the external anal sphincter and pelvic floor muscles, thus correcting pelvic floor dynamics and enhancing coordination between contraction and relaxation. BFT may also improve rectal sensitivity, by recalibrating the sensory threshold, enabling better recognition of bowel movements [11,16-17]. While BFT has shown promising results in adults, as shown by the American Neurogastroenterology and Motility Society and the European Society of Neurogastroenterology and Motility (Level II, Grade B recommendation for short- and long-term FI management), evidence in pediatric populations remains limited, precluding formal recommendations [18].

Rehabilitation programs that combine pelvic floor exercises, electrostimulation, and biofeedback therapy are seldom described in children, with significantly fewer studies available compared to adult research. This can be seen because conducting clinical trials using BFT in children presents unique challenges due to developmental differences, ethical considerations, and logistical barriers. Children's varying cognitive maturity and limited attention spans can hinder their ability to engage with therapy, while the potential anxiety associated with invasive methods like rectal probes may deter participation. Ethical concerns further complicate recruitment, requiring careful oversight to ensure safety and informed consent by the parents. Also, the small sample sizes and the heterogeneity of pediatric populations make it difficult to design standardized protocols, and measuring success is also challenging, as assessments often rely on subjective parental reports or inconsistent self-reporting by children, which can introduce biased information. In addition, maintaining long-term follow-up is logistically demanding, limiting the ability to evaluate sustained benefits. Another possible treatment available is tibial nerve stimulation (TNS). TNS operates by modulating the neural signals transmitted via the tibial nerve, which shares a common origin with the sacral plexus controlling the pelvic floor, anal sphincter, and bladder. This peripheral neuromodulation influences the sacral nerves indirectly, improving the coordination of the muscles involved in continence and regulating bowel reflexes. It can be administered through percutaneous tibial nerve stimulation (PTNS), using a fine needle electrode near the ankle, or transcutaneous tibial nerve stimulation (TTNS), which applies surface electrodes for non-invasive stimulation [17,19]. 

## Review

Methodology 

A literature review on the use of biofeedback therapy (BFT) in children with functional fecal incontinence (FFI) was conducted using electronic databases. This included PubMed, MEDLINE databases, and Google Scholar, focusing on randomized controlled trials and clinical trials conducted. Only articles published in English were considered for inclusion and that were published in the past 24 years. We used the following keywords: "functional fecal incontinence", "functional nonretentive fecal incontinence", "encopresis", "functional constipation", and "biofeedback therapy." Terms were combined using Boolean operators (AND, OR) in order to narrow the search results, guaranteeing a thorough compilation of studies relevant to the research topic. We furthered our research by accessing the selected articles' bibliographic references for inclusion of any potential missing articles.

Inclusion and Exclusion Criteria 

Regarding inclusion and exclusion criteria, in our research, studies were included if they (1) involved pediatric patients (four to 17 years of age) diagnosed with FFI and (2) analyzed the effectiveness of BFT in the treatment of FI in comparison to any other form of treatment or paired to other forms of treatment. Studies were excluded if they (1) were not available in full text, (2) if the intervention treatment did not include BFT, and (3) if the patients suffered from FI with an identifiable organic cause, or had other voiding alterations, such as urinary dysfunction. 

Aim

This literature review aims to compile existing evidence on the use of BFT, either as a standalone treatment or in combination with other interventions, for managing FFI in children. It seeks to evaluate the efficacy and applicability of BFT in pediatric FFI, offering a comprehensive understanding of its role and effectiveness in treatment.

Results 

Study Selection and Data Analyses

From our initial search, 39 articles were retrieved using the outlined search strategy. After removing duplicates, we screened titles and abstracts for relevance and excluded articles that did not meet the inclusion criteria, retaining four articles. Following a citation review of the included studies, one additional article was identified, resulting in a final total of five articles [19-23].

**Figure 1:**
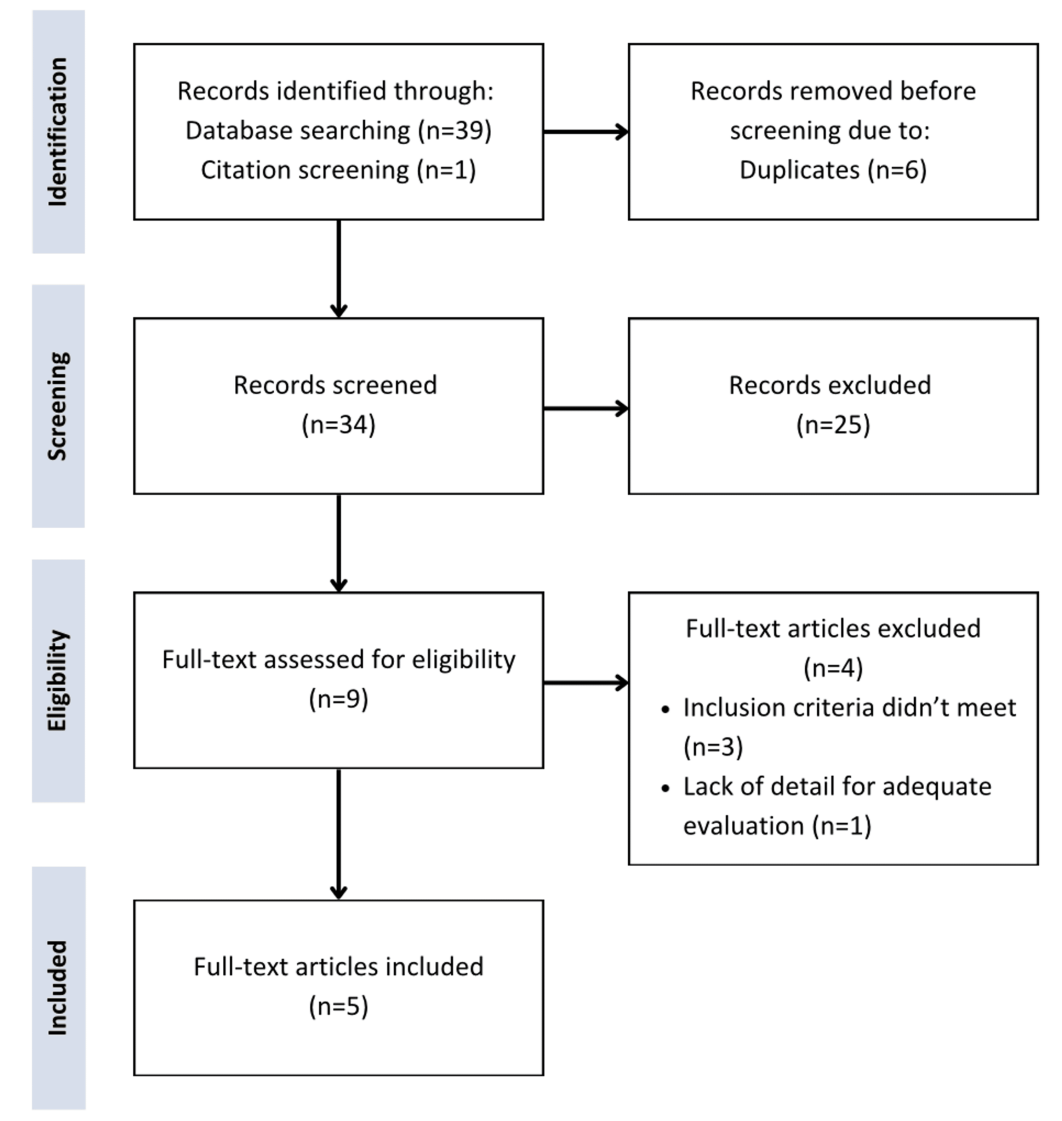
Preferred Reporting Items for Systematic Reviews and Meta-Analyses (PRISMA) flow diagram

Figure 1 illustrates the study selection process. Data from the selected studies were then analyzed based on study design, sample size, age, gender, diagnosis, intervention applied, outcomes, and results.

*Study Characteristics* 

After applying our inclusion and exclusion criteria, a total of five studies were selected for analysis. Of these, three were randomized controlled trials, one was a quasi-experimental study, and one was a retrospective study. The studies included a combined total of 297 patients, all diagnosed with FFI. Two studies focused solely on patients with FNRFI, one on FC, while the other two included patients with both FC and FNRFI. Each study compared a conservative intervention - such as education programs, dietary control, pelvic floor exercise programs, or pharmacological treatment - with the addition of BFT. In three studies, tibial nerve stimulation (TNS) was also evaluated alongside biofeedback, either as a standalone treatment or as an add-on to BFT, resulting in two treatment groups and one control group for these studies. The mean age of participants was 8.73 years. Regarding gender, 63.6% (n = 189) of the participants were male, and 36.4% (n = 108) were female. Follow-up periods ranged from two to twelve months, with most studies conducting follow-ups at three and six months post-intervention. Outcome measures were reported in all studies, although only three differentiated between primary and secondary outcomes. The most common outcome measure was the reduction in FI episodes, although methods of assessment varied. These included the St. Mark’s score, the Rintala continence score, an eight-item symptom questionnaire, the Baylor Continence Scale, and the International Children’s Continence Society (ICCS) criteria. Anorectal manometry parameters were also assessed, including resting and squeeze pressures, rectal sensation thresholds, maximal tolerable rectal volume, and intense urge. One study evaluated quality of life, using the Fecal Incontinence Quality of Life (FI-QoL) questionnaire.

A summary of the included studies’ characteristics is presented in Table 3.

**Table 3:** Summary of study characteristics BFT: biofeedback therapy; ETT: enhanced toilet training; F: female; FC: functional constipation; FI: fecal incontinence; FNRFI: functional nonretentive fecal incontinence; ICC: International Children’s Continence; IMT: intensive medical treatment; KE: Kegel exercises; M: male; PTNS: percutaneous tibial nerve stimulation; NA: not applicable; TFES: transcutaneous functional electrical stimulation; TTNS: transcutaneous tibial nerve stimulation; RCT: randomized controlled trial

Reference	Study type	No. of participants	Mean age	Mean genre	Diagnosis (as described in the study)	Duration of symptoms (in months)	Treatment group (no. of participants)	Control group (no. of participants)	Follow-up (in months)	Outcome measures
Abdelrahman et al. (2021) [20]	RCT	93	9.62 (5-16)	M – 46.3% F – 53.7%	FNRFI	Not reported	B – A + BFT C – A + TTNS	A – Diet + KE	3 and 6m	Incontinence score (St. Marks score); anorectal manometry parameters
Borowitz et al. (2002) [21]	RCT	87	8.53 (6-15)	M – 82.7% F – 17.2%	FI (described as at least one weekly episode of fecal soiling)	> 6m	A – IMT B – IMT + ETT C – IMT + ETT + BFT	NA	14 consecutive days at 3, 6, 12m	Improvement and cure rate
Ladi-Seyedianet al. (2021) [22]	RCT	40	8 (5-12)	M – 72% F – 28%	FNRFI (according to Rome IV)	> 6m	A – TFES + BFT B – TFES	NA	6m	Primary: Incontinence score (ICC scoring system) Secondary: Abdominal pain (VAS), Baylor continence scale, FI-QoL questionnaire
Elshafey et al. (2016) [23]	Quasi-experimental study	54	7.5 (6-8)	M – 53.7% F – 46.2%	Children with weekly episodes of soiling	> 6m	B – A + BFT C – A + PTNS	A – Diet + KE	2m	Rintala incontinence score ; anorcetal manometry
Nader et al. (2020) [24]	Retrospective study	23	10 (7-17)	M – 83% F – 17%	FC (according to ROME IV)	> 12m	Patient and parent education + Laxative treatment + BFT sessions	NA	12m	Rated as a success (no episodes of soiling) or failure

Summary of Outcomes 

The reviewed studies utilized various interventions and methods, with most incorporating multiple study arms and comparing results to a control group receiving conventional treatment. Generally, all studies reported positive outcomes for their interventions compared to controls. However, while most demonstrated a superior outcome in groups receiving BFT, one study found no additional improvement from BFT and another study found that PTNS had slightly better outcomes than BFT. 

Abdelrahman et al. [20] conducted a study with three study arms with the main objective being the assessment of the early effect of BFT versus bilateral transcutaneous tibial nerve stimulation (TTNS) in children with FNRFI who were also receiving conventional treatment. Patients in group A were managed by conventional methods through Kegel exercises (KE) and dietary regulation. Regarding KE, these strengthen the pelvic floor muscles and are performed by the patient. In this case, the child was instructed to lie crock lying position with the knees bent and then instructed to pull his/her pelvic muscles upward and inward and hold the contraction for six seconds, followed by relaxation for six seconds. The exercise was repeated 20 times with a gradual increase in the time until reaching 10 seconds of contraction and relaxation for each with repetition up to 30 times. The exercises were applied twice per week for three months. Besides the conventional methods, group B was managed by BFT (protocol included strength and sensory training, twice weekly for three months using an anal probe) and group C received bilateral TTNS (20-30 minutes and repeated three times per week for three months). Regarding their primary outcome, there was a significant improvement in each individual group at three and six months, but the highest significant decrease was observed in group B. They concluded that biofeedback is more effective than TTNS and that that could be attributed to the ability of biofeedback to increase the awareness of a biological response toward rectal sensation and contraction/relaxation of the external anal sphincter and pelvic floor muscles.

Borowitz et al. [21] conducted a randomized controlled trial using an additive approach in children with FI, hypothesizing that more intensive treatment would yield better outcomes. The study was applied in children with the diagnosis of FI, with no differentiation between FC or FNRFI, although we can assume that the majority had FC considering the treatment employed. Patients were allocated to three groups: group A received intensive medical therapy (IMT), including enemas and laxatives; group B received IMT and enhanced toilet training (ETT) that included a detailed explanation and exemplification by a therapist about how to correctly toilet train; and group C received IMT, ETT, and BFT with surface electromyographic biofeedback to train the external anal sphincter (no information on the number of sessions). Outcomes, measured using an automated patient symptom monitor system (questionnaire with eight questions regarding the patients’ symptoms during 14 days straight at baseline and at three, six, and 12 months), showed that all groups had significant and sustained decreases in the average daily frequency of soiling, but the differences between the three groups were not statistically significant and the same can be said for the cure rate at one year. Group B achieved these results with fewer laxatives and treatment sessions. The authors suggested that even though there was a tendency for better results in group B when selecting a treatment plan for a child with FI, an alternative or additional treatment should be considered if the child does not respond to treatment within two weeks.

Ladi-Seyedian et al. [22] compared the effects of transcutaneous functional electrical stimulation (TFES) alone and TFES with BFT in children with FNRFI. Both groups also received conventional education on pelvic floor function, bowel diaries, and toilet training. After 10 sessions over five weeks, 65% of patients in the TFES+BFT group and 55% in the TFES-only group achieved full continence, with maintained results at a six-month follow-up. BTF was conducted using surface electromyographic biofeedback. While a higher proportion of patients responded to the combined therapy, the difference was not statistically significant. The median of FI frequency per week remained unchanged compared to post-treatment outcomes in both groups and a significant improvement in FI-QoL scores was seen in both groups at six months of follow-up, but with no significant difference between groups. The authors concluded that electrical stimulation, both alone and with BFT, is a reproducible, non-invasive intervention providing symptomatic improvement in children with FNRFI who are refractory to routine conventional treatments.

Elshafey et al. [23] conducted a quasi-experimental study comparing BFT and percutaneous tibial nerve stimulation (PTNS) in children with functional FI. Group A received conventional treatment (daily rectal evacuation, KE, and diet control), Group B received conventional treatment plus BFT (three times weekly for two months using an anal probe for manometric biofeedback), and Group C received conventional treatment plus PTNS (same frequency as BFT). The patients were evaluated at baseline and two months after the interventions using anorectal manometry to measure maximal resting anal pressure, maximal voluntary contraction pressure, voluntary contraction time, the threshold of rectal sensation and maximal tolerable rectal volume and the Rintala continence score to evaluate FI. Anorectal manometry assessments and Rintala scores showed significant improvements in both BFT and PTNS groups compared to the control, with PTNS showing greater improvement across all variables. The study concluded that both PTNS and BFT are effective treatments for FI when added to conventional therapy, with PTNS yielding slightly better clinical outcomes.

Nader et al. [24] retrospectively evaluated biofeedback's impact on defecation urge and sensitivity in 23 patients with FI secondary to FC. Patients attended three monthly consultations, receiving dietary advice and, if needed, medical treatment with laxatives and enemas. In cases with persistent FI, anorectal manometry was used to assess the volume-to-defecation threshold (VTD), and biofeedback sessions (two to five sessions using an anal probe for manometric biofeedback) were recommended based on VTD results. After 12 months, 10 out of 12 children in the success group maintained continence, while two were lost to follow-up. The envy score decreased between the first and last sessions in both groups, with no significant difference between groups. The authors recommended biofeedback for FI cases unresponsive to initial management in children older than seven who can actively participate.

In Table 4, we can see the summary of outcomes and biofeedback program characteristics summarized. 

**Table 4:** Summary of outcomes and biofeedback characteristics BFT: biofeedback therapy; ETT: enhanced toilet training; FC: functional constipation; FI: fecal incontinence; FNRFI: functional nonretentive fecal incontinence; KE: Kegel exercises; PTNS: percutaneous tibial nerve stimulation; TFES: transcutaneous functional electrical stimulation; TTNS: transcutaneous tibial nerve stimulation; VTD: volume-to-defecation threshold

Reference	Type of biofeedback	Number of sessions	Results	Conclusions
Abdelrahman et al. (2021) [20]	Anorectal manometry-based biofeedback	Twice weekly for 3 months(~24 sessions)	BFT is more effective than TTNS for short-term FNRFI improvement	BFT superior to TTNS, KE and diet for FNRFI in short-term follow-up
Borowitz et al (2002) [21]	External anal sphincter electromyographic biofeedback	Not specified	ETT is most effective for reducing soiling frequency, BFT beneficial but not superior	ETT highly effective, BFT aids but does not surpass behavioral approach
Ladi-Seyedian et al (2021) [22]	External anal sphincterelectromyographic biofeedback	Twice a week, 10 sessions	Higher FI improvement rates with TFES + BFT	BFT enhances TFES effectiveness, useful in combined treatment
Elshafey et al (2016) [23]	Anorectal manometry-based biofeedback	Twice weekly for 2 months(~16 sessions)	PTNS slightly more effective than BFT, both improve anorectal function and incontinence scores	PTNS preferable, but BFT remains valuable especially when PTNS is unviable
Nader et al (2020) [24]	Anorectal manometry-based biofeedback	Average 3 sessions (range 1-5)	BFT effective in reducing VTD, sensory response improved in resistant FI cases	BFT beneficial in FC cases resistant to other treatments

Discussion 

In cases of FFI, the first line of treatment typically involves non-pharmacological, non-invasive approaches as we have already mentioned. Additional treatments, such as BFT, are considered when initial measures fail, although their clinical relevance remains uncertain due to limited supporting evidence [2,3]. We also saw in recent studies that by the age of 18, 15% of the children with FFI still suffered from FFI problems [2]. This leads us to believe that a multimodal rehabilitation approach, incorporating conventional treatments with BFT or electrical stimulation, might be beneficial from the outset, as it is done in the adult population. This review aimed to evaluate the effectiveness of BFT in pediatric FFI, considering it is a low-risk intervention with generally positive outcomes in adults. 

Our search identified five studies on BFT for pediatric FFI, highlighting the limited scope of research in this area. Although most studies shared similarities in population characteristics (e.g., age and gender distribution), there were notable limitations and methodological differences. Three studies were randomized controlled trials, each with distinct methodologies, while the remaining studies were quasi-experimental and retrospective studies.

Diagnoses also varied, with two studies focused on patients with FNRFI, two on FC, and one without differentiation between FNRFI and FC. Although Borowitz et al. did not use the Rome IV criteria to specify the type of FI, we assume that the patients had FC due to the type of treatment that was applied (fecal enemas for fecal impaction and laxatives). 

Regarding the BFT protocols, its use varied widely across studies, which may explain some discrepancies in outcomes. For instance, Abdelrahman et al., Elshafey et al., and Nader et al. used anorectal manometry-based biofeedback, while Borowitz et al. and Ladi-Sevedian et al. used an electromyographic biofeedback with surface electrodes applied near the external anal sphincter. All of the studies had different numbers and duration of sessions, which adds to the discrepancy between the studies. Both methods, manometric or electromyographic biofeedback, have their role in the management of FFI. Manometric biofeedback using a rectal probe provides direct and precise muscle activity measurements but is more invasive, which can cause discomfort, especially in younger patients. Surface electrodes offer a less invasive, more comfortable option but may yield less specific data due to potential interference from surrounding muscles, like the gluteus muscles. In pediatric populations, surface electrodes are often preferable to minimize anxiety and discomfort. However, no studies to date directly compare the efficacy of manometric versus electromyographic biofeedback in FFI treatment or their tolerability amongst the pediatric population. The number of BFT sessions also varied, as there is no standardized protocol, even in adult populations. Typically, the number of sessions is adjusted based on individual patient response and clinical progress. 

In terms of clinical outcomes, all studies incorporated conventional treatment (e.g., education, bowel diary, toilet training, and pelvic floor exercises, but the specificities of each treatment were not described in detail) as a baseline intervention.

Analyzing the selected studies regarding the type of FI, two studies, Abdelrahman et al. and Ladi-Sevedian et al., two large RCTs, focused solemnly on patients with FNRFI. The first, besides being the largest RCT in our review, concluded that biofeedback is more effective than TTNS and that that could be attributed to the ability of biofeedback to increase the awareness of a biological response towards rectal sensation and contraction/relaxation of the external anal sphincter and pelvic floor muscles. The second, also an RCT, showed that BFT could aid TFES with higher FI improvement when used in combination. Regarding the type of BFT, both had different protocols, with Abdelrahman et al. using anorectal manometry and Ladi-Sevedian et al. electromyographic. In the first case, the mean age was slightly higher (9.62 vs. 8), which means that the patients could be more comprehensive toward the use of a rectal probe. Both studies conclude that the use of BFT should be used in FNRFI refractory to conventional treatment.

Regarding FC, we had two studies, Nader et al. and Borowitz et al. The first conducted a retrospective study that showed positive results in the use of BFT in refractory cases. The second did not show any additional value in the use of BFT. Comparing both studies, we can see that in the first case, all the patients had a total of five sessions and used anorectal manometry, and the second one did not report the number of sessions applied and used electromyographic BFT. Besides these results, in both studies, they conclude that when selecting a treatment plan for a child with FFI, an alternative or additional treatment with BFT should be considered.

Finally, in the quasi-experimental study of Elshafey et al., they did not differ between FC or FNRFI, and no pharmacological treatment was applied, so in this case, it is not possible to assume which type of FI was predominant. On the subject of the study, they compared the difference between BFT and PTNS in treating functional FFI in children and concluded that both PTNS and biofeedback are effective modalities in treating children with FFI in addition to conventional treatment with better results regarding the use of PTNS. They used anorectal manometry with a total of 16 sessions and used percutaneous rather than transcutaneous, which could be hard to employ in younger children. 

An important aspect of BFT is its safety profile, as no adverse effects were reported in any of the studies reviewed, beyond the potential discomfort associated with rectal probes. However, most studies did not systematically document patient tolerance or comfort.

Limitations

This review highlights several challenges in studying BFT for children with FFI, limiting the strength of current evidence. As already described, developmental differences, anxiety related to invasive methods, and ethical concerns complicate participation and recruitment. The small sample sizes and the heterogeneity of pediatric populations also reduce statistical power. In addition, the lack of standardized BFT protocols for children means that each hospital often employs its own methods, further contributing to variability in outcomes. Reliance on subjective parental reports and logistical difficulties in maintaining long-term follow-up also hinder assessments of sustained benefits. These limitations emphasize the need for standardized protocols and more rigorous studies to optimize BFT for pediatric populations.

## Conclusions

BFT is an adjunct to conventional therapy in refractory cases in FFI, and it is a viable option for both FC and FNRFI. In conclusion, BFT represents a promising non-invasive treatment for pediatric FFI. When used in a tailored, multimodal approach, it holds the potential to improve continence and quality of life in children with this challenging condition, and given that 15% of children with FFI, specifically FNFRI, continue to experience symptoms into adulthood, it is crucial to consider these treatment options early to potentially reduce this rate. Further well-designed, large-scale studies are warranted to confirm its long-term benefits and to refine biofeedback protocols for more consistent clinical application.
